# New York Medico-Chirurgical Society—Tetanus, &c.

**Published:** 1856-11

**Authors:** J. O. Bronson

**Affiliations:** Secretary


### SELECTIONS.

*New York Medico-Chirurgical Society.* Reported for the *American Medical Monthly*, by J. O. Bronson, M.D., Secretary.

*July 8th.*—The following case of Tetanus, was related by Dr. Benjamin Lee:—

Solomon Jackson, aged 39, entered hospital June 3d, 1856, with tetanus, the result of a large sloughing ulcer on the inside of right leg. The spasms involved the entire system, and were purely opisthotonic. Felt less in left leg than elsewhere. Could separate the teeth about an eighth of an inch. Was unable to protrude the tongue, which was badly bitten, and to chew his food. Was very weak; pulse 130, weak and irritable. Tone of system, poor. Has had no evacuation for four days; sleepless; spasms occurred about every three minutes.

He gave the following history:—In January last, a small tumor made its appearance at about the centre of the present site of the ulcer, which gradually increased till it burst. The ulcer, thus made, spread with considerable rapidity. It was treated with poultices and salves, until about six weeks since, when he consulted a botanical doctor, who pronounced the sore to have been caused by the bite of a tree toad! and proceeded to cut away portions of it—whether merely to remove sloughs, or with a view to "cutting out the poison," I am not able to say. He also gave him an "Improved Healing Salve," and an "Improved Healing and Drawing Salve."

No benefit was derived from these applications; and, on the evening of Saturday, May 24th, the patient began to feel cramps, starting from the leg and running up to the jaw. These gradually increased to their greatest intensity. The patient's countenance wore an expression of extreme anxiety. The following was prescribed, and a table-spoonful given every second hour:—

℞—Spt. Vin. Gal. . . f℥ij.

Cinchonæ pulv. . . . . ℥j.

Aq. puræ, . . . . f℥iv.

M.

Also,

℞—Calomelas, . . . . . gr.x.
Gambog. pulv. . . gr.iij.
Ft. pil. no. 1,

Given at once.

And,

℞—Gum. Opii, } aa ℈j

Ext. Conii, }

Ft. pil. no. 10; of which one was given every two hours.

A large bread poultice, with tr. opii, ʒj. and aq. ℥j. was applied to the ulcer. This was at 3 P.M. At 7—the leg feeling very painful, and as if the poultice scalded it—substituted warm water dressings. 12 P.M. condition about the same; leg comfortable. Sleepless. Substituted for the opium and conium, the following:—

℞—Ext. Cannab. ind. . . ʒss

Div. in pil. no. 10, and gave one every two hours.

*June 4th, A.M.*—Pulse 98; has had some refreshing sleep since taking the cannab. ind. Great tenderness and distressing spasms of diaphragm, and in right iliac region. Applied to both, warm fomentations of fol. aconit. No evacuation. Gave,

℞—Ol. Ricini, . . . ℥iss.

Ol. Tiglii, . . . . gtt.ij.

Increased the brandy and cinchona mixture to ℥iij. Continued the cannab. ind. as before.

*P.M.*—Has vomited a large quantity of yellow, curdy matter, probably milk, which I had ordered him to take freely. Added a little calc. chl. to the dressing for the ulcer. Leg comfortable; spasms less frequent, but very violent, almost throwing him from the bed.

*June 5th.*—Retention of urine; had passed no water since the previous night. Pain above pubes. Applied a moist warm fomentation of hops over bladder. Prescribed,

℞—Pulv. Opii, } aa gr.iij.
" G. Camph. }
Ft. pil. no. 3; two to be given immediately, and one in an hour. No evacuation. Dispirited. Pulse more frequent and irritable. Mist. cinchon. was given every hour. Spasms a little less frequent. 10 P.M. had passed water with considerable difficulty. Gave,

℞—Hyoscyam. pulv. } aa gr.ij.

Opii, }

Ft. pil. no. 2.

*June 6th, A.M.*—Has had a large soft evacuation. Spasms diminished in frequency and violence; more confined to legs. Less trismus. Has not bitten his tongue for two nights. Still considerable rigidity of muscles of mastication. Leg painful—probably owing to a change in the temperature. Increased the calc. chl. in the water dressing. Ulcer dark and foul in spots. Pulse 100; urination much easier; slept well.

10 *P.M.*—Pulse 84, full and round; leg comfortable; spasms less frequent, most severe in abductors of thighs, jerking the legs wide apart—very slight in upper extremities or trunk. Bowels quite loose; four evacuations during the day, unattended with pain; micturition easy.

*June 9th, P.M.*—Since last record the treatment has been as before. Yesterday, diminished the dose of cannab. ind. to gr. ij. every two hours. Ulcer generally clear, suppurating, and granulating. Spasms now felt only at knees. They appear to be principally in the vastus internus and sartorious—feeling, as he says, as if they were dragging his knees together. They do not occur now more frequently than every ten minutes. Can protrude the tongue, if done carefully, without inducing them. Ate a little softened bread, to-day, with some comfort. Bowels free, owing probably to the cinchona. Pulse 83, full and soft. Countenance still anxious.

*June 10th.*—Has slept well; spasms slight, not oftener than once in fifteen minutes, induced by a sudden noise. Pulse 80. Alternated the cannab. ind. with opii et camph. aa, gr. ij. to be given every two hours.

*June 15th.*—Improvement still continued. Altered treatment as follows:—

℞—Cannab. ind. ext. gr.xxx.
Sulph. fer. exsic. gr.ix. M.
Div. in pill no. 30.

Ol. Jec. Asel. A table-spoonful three times a day.

Since that time his improvement has been steady. He is now able to masticate his food without trouble. For a slight twitching in the sole of the foot, I, to-day, applied a fomentation of fol. aconiti, which afforded almost immediate relief. The ulcer is still, however, in an unhealthy state, and he is liable to a relapse into his tetanic state. But I think, that as far as that attack of the disease goes, he may be considered cured.

In answer to an inquiry, Dr. Lee remarked, that no diuretic effects were observed resulting from the use of the cannabis indica.

Dr. Carnochan spoke of several cases of tetanus, which had passed under his observation, in one of which the inefficiency of chloroform, as a remedial agent, was fully demonstrated. At least half a pound of the article was used with no other effect than to allay the spasmodic action for a time. As soon as its use was discontinued, the paroxysms appeared with equal if not renewed intensity, until at last the patient died. Recovery from true tetanus was a rare occurrence. He had seen some cases simulating tetanus, wherein medicine was effectual. In one, wine and the carbonate of iron produced a cessation of the spasms. Sometimes a removal of the cause is productive of the happiest effects. Being called to see a child, who, in running about barefoot, had pierced the sole of his foot with boxwood stubble, he found the patient suffering extremely with trismus, which had continued for five or six days. Suspecting the presence of a portion of the stubble in the bottom of the wound, he cut down upon and removed a small piece, which had been the exciting and continuing cause of all the disturbance. The child immediately began to convalesce. As regarded the case related by Dr. Lee, he remarked that he had never known a case of traumatic tetanus to occur after the twenty-first day from the reception of the injury; and, if tetanus is liable to be produced by an ulcer, the case was certainly interesting.

Dr. Carnochan then exhibited two large and several small pieces of the frontal bone, together covering a surface of six and a half square inches, removed from a man who had suffered a comminuted fracture by a falling bar of iron. The fracture involved a portion of the orbit, and infringed upon the parietal bone of the right side, wounding the branches of the middle meningeal artery. The injury occurred eight days before. At first, the man was stunned by the blow, but, after forty-eight hours, he had been sensible and able to answer intelligibly. At the time of the operation for the removal of the bone, the man was inclined to sleep. The pupils of the eyes were not dilated. There was no febrile excitement, and not much sign of compression. His pulse was 45. With slight effort the pieces of bone were removed with polypus forceps, no trephining being necessary. A coagulum of blood was found extending under the sound bone, farther than could be reached. All was removed, however, that was possible, and the parts gently washed by means of a sponge. No pus had formed, although eight days had elapsed since the injury. Immediately after the removal of the bone, the pulse arose to 80. The prognosis was doubtful.

Dr. Bouton presented the stomach of a woman, who, tired of a life spent in drunkenness and prostitution, had taken arsenic, which was followed by death. The organ was presented, however, more to exhibit an abnormal condition or formation at its cardiac end, showing an irregular cyst, puffed up with air, appearing as if the inner coats of the stomach had suffered solution of continuity at some time, and the remaining tissues had become dilated into a pouch, capable of holding about two drachms. After examining the organ, Dr. Cox remarked, that the appearances internally would indicate that an ulcer had existed at that point; and he was of the opinion that such was the cause of the condition.

Dr. Cox presented, for Dr. Leigh, of the Nursery Hospital, a heart removed from a girl, aged 12 years, weighing eleven ounces, about twice the size of the organ in a healthy condition. Last winter, as was reported at the time of her entrance into the Hospital, she was affected with pains in her limbs and joints, which were considered by the physician in attendance as "growing pains." The joints were also swelled, and she probably had endocarditis, associated with rheumatism. She was admitted into the hospital on the 18th of June, suffering dyspnœa. Her pulse was rapid, and the action of the heart was violent. The sounds were blended and gave a bellows murmur. The patient was anæmic and emaciated. The face became œdematous and the feet swelled. Digitalis was temporarily beneficial in controlling the action of the heart. Gastritis became developed and diarrhœa supervened, which was uncontrollable, on account of the irritability of the stomach. Enemata and suppositories were used without effect. The evacuations, which were at first of a dark color, became mucus and blood. Violent pain existed in the gastric region. About two days before death, the heart's action became more natural. No delirium occurred at any period of the disease.

*Post Mortem.*—Examination, post mortem, found the lungs slightly congested; the stomach inflamed; the spleen enlarged to three times its natural size; the liver also hypertrophied, and some effusion into the cavity of the peritoneum. The heart (which is spoken of lastly, it being before the Society,) was found very much diseased. Its size, as mentioned before, was nearly doubled, the left ventricle dilated, and the auricle of the same side showed evident signs of inflammation. The mitral valves, also, presented some evidences of inflammation having existed.

Dr. Cox also presented a portion of the ilium and colon of a child, one year and two months old, who but a few days before was apparently well. The bowels were regular, appetite good, and no evidence of disease existed. It had been weaned four months. The first symptom was a slight diarrhœa. The gums were scarified, as they were red, swollen, and tender. The number of stools did not exceed three in the twenty-four hours, which were thin and bilious in their character. After the first day, the stools were more thin than at first, with less fæcal matter. Until within eight hours of its death, there were no symptoms of sinking or prostration, the pulse was rapid, and the child's face, the afternoon before its death, wore an expression of suffering without any other evidence. There was no distension of the abdomen, and the child, throughout, rested quietly. A prescription, containing camphor-mixture, with two drachms of chalk-mixture to the ounce, had been prepared for it, a spoonful of which, once in six hours, had been given. It did not nurse as well, or as much as usual, the last two days.

There had been no vomiting until six hours before death, when it commenced to vomit, and cry as if suffering severe pain; the feet were drawn up, accompanied by two or three green evacuations, with spasmodic twitchings of the muscles of both extremities. Warm fomentations were applied to the abdomen, and a quarter of a drop of Magendie's solution of morphia was administered; after which it rested quietly for two hours, sleeping at intervals. After this, the same symptoms returned; the mixture was repeated, but did not produce sleep. The little patient continued to sink, and died in a few hours. The specimen presents the glands of Peyer enlarged and inflamed; the solitary glands are slightly ulcerated, and the whole of the colon is very much inflamed. The Doctor remarked, that he did not remember having seen an instance where so much disease had existed, in a child, with so few symptoms.

